# Personality Traits and Annual Income Determine the Willingness to Pay for a Single Tooth Implant

**DOI:** 10.3390/healthcare9080952

**Published:** 2021-07-29

**Authors:** Shirlene Foo Yih Ting, Kimberley Chew Wen Chien, Nurul Hanis Ramzi, Allan Pau, Rohit Kunnath Menon

**Affiliations:** 1 School of Dentistry, International Medical University, Kuala Lumpur 57000, Malaysia; SHIRLENE.FOOYIH@student.imu.edu.my (S.F.Y.T.); KIMBERLEY.CHEW@student.imu.edu.my (K.C.W.C.); allan_pau@imu.edu.my (A.P.); 2 Institute for Research, Development and Innovation, International Medical University, Kuala Lumpur 57000, Malaysia; nurulhanis@imu.edu.my; 3 Restorative Dentistry, School of Dentistry, International Medical University, Kuala Lumpur 57000, Malaysia

**Keywords:** willingness to pay, dental implant, personality, cost, single tooth implant

## Abstract

The objective of this study was to evaluate the factors influencing the willingness to pay for a single tooth implant in Malaysia and to assess if an additional evidence-based patient education video increases the willingness to pay. A total of 100 subjects seeking single tooth replacement at the Oral Health Centre, International Medical University (IMU), Kuala Lumpur, Malaysia, were asked to complete questionnaires about personal demographics and personality traits. Subsequently, they were randomly allocated into two groups. Group C received a conventional patient–dentist interaction on treatment options for missing teeth, while Group EV received the same content with an additional evidence-based video on the survival rate and complications for each option from recent meta-analyses. Willingness to pay the median price and the highest price that the subjects were willing to pay were assessed by a structured bidding process. A higher annual income was significantly associated with willingness to pay the median price for a single tooth implant (χ^2^ = 6.91, *p* = 0.03). Dominant personality traits of openness (r = −0.25), conscientiousness (r = −0.30) and agreeableness (r = −0.20) were negatively correlated with the highest price that the patients were willing to pay for a single tooth implant (Pearson’s correlation test, *p* < 0.05). No significant difference in willingness to pay was found between Group C and Group EV (χ^2^ = 0.05, *p* > 0.05). In conclusion, patient education strategies for single tooth replacements with dental implants should be customized based on a patient’s personality and income to maximize effectiveness.

## 1. Introduction

Dental implants have a decisive advantage as compared to a tooth-supported and removable prosthesis for replacement of a single missing tooth [1]. However, a higher cost limits the utilization of this treatment modality by a wide spectrum of the population [2]. Therefore, lower implant treatment costs and appropriate pricing of dental implants plays an important role in increasing the number of patients who might opt for a dental implant to replace missing teeth. The cost of implant treatment should ideally be well-aligned with the willingness to pay for the treatment in order to maximize the benefits of the treatment at a community level.

Willingness to pay (WTP) refers to the maximum amount in monetary terms that an individual would be willing to sacrifice in order to obtain the benefits of a treatment [3]. WTP assessment allows for a direct cost–benefit analysis and can serve as a guide in terms of pricing and demand forecasts for individual healthcare services [4,5]. Gender, annual income, educational level, professional qualifications and working situation were previously found to influence the willingness to pay for healthcare services [6,7]. Further, personality factors such as social character, compliance, aggressiveness, ethnocentrism and dogmatism were also found to affect consumer buying behaviors [8]. Hence, assessment of different factors including personality traits is necessary to understand the factors associated with the willingness to pay for a healthcare service. However, WTP assessments are subjective and are also susceptible to bias [9]. Therefore, studies with a robust methodology are required to minimize the factors contributing to the bias. Patients must understand the benefits of a treatment comprehensively for an accurate assessment of the willingness to pay for that treatment. It was previously found that improving the content validity of the information provided to the patient can ensure that patients clearly understand the treatment benefits, thus increasing the willingness to pay a higher price for the treatment [10]. Improving the content validity, when combined with an appropriate sampling strategy, may ensure adequate long-term stability for the WTP measures [11].

Even though numerous studies have evaluated the WTP for various kinds of dental treatment [12], studies assessing WTP for dental implant treatment are not many and seem to focus more on willingness to pay for implant overdentures than single tooth replacement [13,14,15,16,17,18]. Previous research established that the gender and annual income of an individual have a significant influence on the willingness to pay for dental treatment [6,7]. However, the willingness to pay for dental treatment may also be influenced by additional factors, including the personality of the patient and also the patient’s knowledge level concerning the treatment being offered. We hypothesized that increasing the content validity of patient education, by providing an additional evidence-based video, may enhance the understanding of treatment benefits for dental implant therapy, resulting in willingness to pay a higher price.

Our study aimed to assess the factors influencing the willingness to pay for dental implant treatment, and to compare the willingness to pay for a single tooth dental implant in patients who received an additional evidence-based video and in patients who received a conventional one–one verbal interaction on treatment options with a dentist.

## 2. Materials and Methods

Ethical approval for the study was obtained from the Joint Committee on Research and Ethics at the International Medical University, Kuala Lumpur, Malaysia, and the study was conducted as per the guidelines specified by the Institute of Research, Development and Innovation (IRDI), International Medical University, Kuala Lumpur, Malaysia.

A survey was carried out in 25 dental clinics in the Klang Valley, Kuala Lumpur, Malaysia, to identify the median price for replacement of a single missing tooth with a dental implant.

The study was conducted at the Oral Health Centre (OHC), International Medical University (IMU), Kuala Lumpur, Malaysia. Patients with missing teeth who attended the OHC were recruited for the study based on the following selection criteria:

Inclusion criteria:

1. Adults >18 years who were employed and were the primary decision makers for the payment for treatment;
2. Subjects who gave a written informed consent;
3. Subjects having at least one missing tooth;
4. Subjects opting for an out-of-pocket payment mode;
5. Subjects considering replacement of the missing tooth with one of the available options in the clinic;
6. Subjects who could read and understand English.

Exclusion criteria:

1. Subjects who were not the primary decision makers in payment for treatment (spouse, children, dependents);
2. Subjects who were insured for payment for dental treatment;
3. Subjects who were participating in any clinical trial;
4. Subjects who were satisfied with the current dentition and were not thinking of replacement of the missing teeth;
5. Subjects who had previously undergone implant treatment;
6. Subjects who could not read and understand English.

Eligible participants were provided with a study information sheet that described the study methodology. The subjects were given one week to consider joining the study and provide a written informed consent.

The power of the sample was calculated using Epi Info^TM^ based on the minimum sample required for comparing the two groups with a single dependent variable (WTP the price of a single median implant), with a two-sided confidence interval of 95% and power of 80%. Based on an anticipated intervention effect of at least 30%, assuming a proportion of 60% patients to be willing to pay the median price of an implant when given the conventional one–one verbal interaction session, the required sample size was calculated to be 50 per group.

Data collection:

We recruited 100 subjects and provided them with a previously validated questionnaire to collect data on age, gender, basic education, professional training, working situation, professional status, annual income and previous dental visits (Supplementary material, Table S1)

Following this, the recruited subjects were asked to complete a second questionnaire to assess their personality. We utilized the OCEAN (Openness, Conscientiousness, Extraversion, Agreeableness and Neuroticism) Model to evaluate the personality traits of each subject (Supplementary material, Table S2) [19].

Subsequently, subjects were randomly allocated into two groups by a computer-generated random allocation method [20]. One dentist (KCWC) imparted awareness about treatment options and benefits to both groups. Group C (Conventional, n = 50) underwent an 8-min one–one interactive session with the dentist in which the dentist explained the treatment benefits of dental implants and also alternative options (fixed-fixed bridge, cantilever bridge, resin-bonded bridge and removable partial denture) while giving the subject an opportunity to ask questions. Models and photographs were used when explaining all five treatment options.

Group EV (additional Evidence–based Video, n = 50) underwent an 8-min interaction with the same dentist and were provided the five treatment options by using the same models and photographs. In addition to this, a video that provided information about the survival rates and complication rates for each of the available treatment options was shared. The video was prepared by KCWC and SFYT based on the most recent evidence from systematic review and meta-analyses [21,22,23]. Subsequently, the video was reviewed by RKM. The video was of 2-min duration and the subjects were allowed to ask questions during the video in case they had any queries.

**Figure 1:**
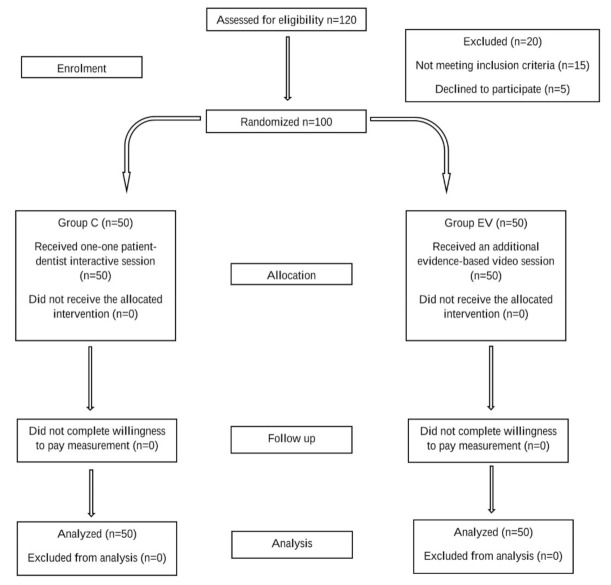
Study flowchart.

Another researcher who was blinded to the previous interactive sessions assessed the willingness to pay. A bidding process was administered to the subjects in a two-stage process. In the first stage, subjects in each group were asked if they were willing to pay the median price of a single tooth implant in Kuala Lumpur (calculated from the pilot survey of 25 dental clinics in Klang valley, Kuala Lumpur, Malaysia). In the second phase, for subjects who were not willing to pay the median price, the price was progressively reduced by RM 500 (USD 120) until it reached a price that the subjects were willing to pay or when the sum reached 0. Subjects who were willing to pay the median price for an implant were asked if they were willing to pay a still higher price. The price was progressively increased by RM 500 (USD 120) until the subject was no longer willing to pay a higher price or until the maximum price for a dental implant in Kuala Lumpur was reached. The study flow is summarized in Figure 1.

Statistical analysis

The data gathered were analyzed using the Statistical Package for Social Sciences software (SPSS for Windows, version 25.0, 2004, Chicago, IL, USA).

Pearson’s chi-squared test (χ^2^) was used to compare the sociodemographic factors and the willingness to pay the median price for a single tooth implant. Multiple analysis of variance (MANOVA) was used to determine if the personality traits influenced the willingness to pay the median price for a single tooth implant. Pearson’s chi-squared test (χ^2^) was used to compare the willingness to pay the median price between the two groups. Analysis of variance (ANOVA) was used to compare the sociodemographic factors and the highest price that the subjects were willing to pay for a single tooth implant. Pearson’s correlation test was used to analyse the association between the five-factor personality constructs and the highest price that the subjects were willing to pay. The mean price for a single tooth implant that each group (C and EV) was willing to pay was compared using ANOVA. A maximum permissible type I error α = 0.05 was adopted, whereas *p* ≤ 0.05 was considered statistically significant.

## 3. Results

The demographic characteristics of the two groups were closely matched in terms of gender, education level, professional status and annual income levels (Table 1).

The median price for replacement of a single missing tooth with a dental implant calculated from our survey of 25 dental clinics in Kuala Lumpur was 6000 Ringgit Malaysia (RM) (USD 1400). The minimum price from the survey was RM 5500 (USD 1300), and the maximum was RM 9500 (USD 2300) (Supplementary material, Table S3). Our results show that only 30 subjects were willing to pay the median price of RM 6000 (USD 1400) for replacement of a single tooth with a dental implant. Annual income had a significant impact on the willingness to pay the median price for a single tooth implant (χ^2^(2,100) = 6.91, *p* = 0.03) (Table 2). The personality trait of conscientiousness was significantly associated with willingness to pay the median price of a single tooth implant (F (1,100) = 6.84, adjusted *p* = 0.01) (Supplementary material, Table S4). All remaining sociodemographic and personality traits did not have a significant influence on the willingness to pay the median price for a single tooth implant. There was no significant difference in the proportion of subjects who were willing to pay the median price when comparing Group C to Group EV (χ^2^(1,100) = 0.05, *p* = 0.82) (Table 2).

From the data on the highest price that the patients were willing to pay for a single tooth implant during the bidding process, the mean price was calculated for each group (Supplementary material Table S5). None of the sociodemographic factors had a significant influence on the highest price that subjects were willing to pay (ANOVA model, *p* > 0.05). However, Pearson’s correlation test indicated that personality traits of openness, conscientiousness and agreeableness were significantly associated with the highest price that the subjects were willing to pay for a single tooth implant (Table 3).

**Figure 2:**
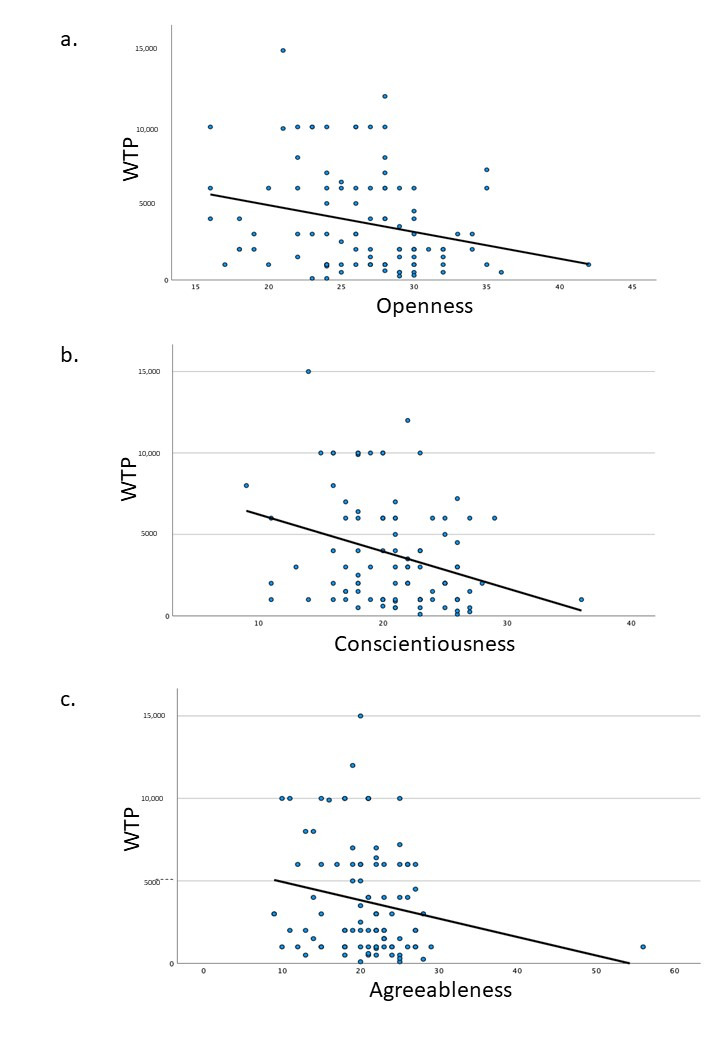
Correlation of personality traits to willingness to pay. (**a**) Openness, (**b**) Conscientiousness, (**c**) Agreeableness.

As shown in Figure 2, the personality traits of openness, conscientiousness and agreeableness exhibited a negative correlation to the willingness to pay. Age was also negatively correlated to agreeableness (r = −0.26, *p* < 0.001). None of the other factors exhibited a significant influence on the willingness to pay for a single tooth implant.

The mean price that Group C was willing to pay for a single tooth implant was RM 3699 (USD 880) (±3448), while Group EV was willing to pay a mean price of RM 3766 (USD 900) (±3171). There was no significant difference between the two groups when comparing the highest price they were willing to pay for a single tooth implant (F (1,100) = 0.31, *p* = 0.577).

## 4. Discussion

We utilized a bidding process to elicit the highest price that the patients were willing to pay for a single tooth implant. Bidding is a widely accepted method to assess willingness to pay, especially for those unfamiliar with healthcare payments [24]. However, a bias may be generated on the starting amount, which was eliminated by the use of the median price as recommended by previous research [25].

The median price for a dental implant in Kuala Lumpur calculated from our survey was RM 6000 (approximately USD 1400). The result was similar to a recent survey on worldwide dental implant prices [26]. The survey reported a price of USD 1500 in Malaysia. The median price for a single tooth replacement in Malaysia was much lower than that in Singapore (USD 2700) and the United States (USD 2500). The prices were comparable to Thailand (USD 1720), South Korea (USD 1350), Israel (USD 1200), Columbia (USD 1200) and Turkey (USD 1100). Lower prices for dental implant treatment were reported in Poland (USD 925), India (USD 900), Mexico (USD 900), Jordan (USD 900) and Costa Rica (USD 800). Only 30% of the participants were willing to pay the median price for a single tooth implant in our study. This may be explained by the fact that the median price (RM 6000) (USD 1400) is higher than the median household income in Malaysia [27]. The best treatment option for a single missing tooth does not seem to be affordable for a majority of the participants. Dental healthcare providers need to align the willingness to pay for treatment with the cost in order to provide the maximum benefit to society and hence promote responsible profiting. The quality of dental implant treatment should not be compromised either. Hence, patient education should be aimed at enhancing awareness about the benefits of dental implant treatment, and a middle ground has to be attained.

Our results show that age and gender do not significantly affect the willingness to pay for a single tooth implant. This result is similar to a previous study conducted in Saudi Arabia on the willingness to pay for dental implants [6]. In contrast, a study conducted on Finnish adults implicated age and gender with variations in the willingness to pay for unexpected dental expenses [7]. However, implant treatment is not an unexpected dental expense and hence might explain the difference in the results when compared to our study. In agreement with the Finnish study [7], a higher annual income was associated with willingness to pay the median price for a single tooth implant. There is previous evidence on the influence of a positive association of income on the willingness to pay [28,29,30]. Another study that investigated the willingness to pay for dental implant treatment in Hong Kong cited gender and educational attainment as significant factors associated with the willingness to pay, with income not mentioned as a factor [31]. The aforementioned results quite interestingly suggest an international variation in factors determining the willingness to pay and may suggest the involvement of additional factors or variables such as different personality traits, which may be dissimilar in different nations.

We utilized the OCEAN (Openness, Conscientiousness, Extraversion, Agreeableness and Neuroticism) Model to evaluate the personality traits of each subject [19]. This model has been an innovative and ground-breaking concept that helps to identify the rare, exceptional and unusual characteristics of an individual. A previous study has confirmed the validity and reliability of the Big Five Inventory in Malaysia [32]. Our research shows that personality traits generally do not influence the binary decision of whether to pay or not to pay the median price of a dental implant, except for the trait of conscientiousness. Patients with a dominant trait of conscientiousness are less willing to pay the median price for a single tooth implant. Further, our results show that conscientiousness is also negatively correlated with the highest price subjects are willing to pay. In addition, openness and agreeableness are also negatively correlated with the highest price subjects are willingness to pay for a single tooth implant. Conscientiousness is associated with good impulse control, organized behavior and mindfulness of details. Openness is associated with willingness to try new things, whereas agreeableness is associated with cooperative and pro-social behavior [19]. Good impulse control and higher cooperative mentality associated with conscientiousness and agreeableness may explain the negative correlation of these traits with willingness to pay for dental implants and openness of the patients to other treatment modalities. A previous study on impulsive buying behavior stated that openness, extraversion and neuroticism had a positive impact on impulsive buying behavior [33]. This implies that the combination of openness with a more aggressive personality trait is associated with impulse buying. However, the decision to undergo dental implant treatment involves making a decision after understanding the benefits and risks of a procedure and cannot be considered impulsive buying, hence explaining the difference in the results.

We found no significant difference in the proportion of subjects who are willing to pay the median price of RM 6000 for a single tooth implant when comparing the group who underwent the conventional interaction with the dentist to the group who received the additional video intervention. Further, the two groups showed no difference in the highest price they were willing to pay when compared to each other. Our results show that enhancing content validity by giving an additional evidence-based video did not significantly increase the willingness to pay for dental implant treatment. Willingness to pay for dental implant treatment in Malaysia seems to be influenced by the annual income and personality traits of an individual. There is previous evidence that dentists may propose treatment options to patients based on their appearance and demographic details alone, especially withholding or suggesting high cost treatment options such as dental implants [34]. This approach may be unethical as it may lead to unneeded treatment, inability for patients to participate in the decision-making process and the undermining of healthcare for business gain. Informed decision making is an essential component of ethical healthcare delivery. Hence, we propose that understanding and analyzing the individual patient factors should be given significant importance when deciding the price and designing the educational content for dental implant treatment in Malaysia.

Missing values and protest zeros may pose a problem in contingent valuation studies that assess the maximum and minimum value that a subject is willing to pay for a commodity [35]. This may be attributed to an incomplete understanding of the proposed question or due to reluctance to answer. However, no missing values and protest zeros were reported in our study. The results of our study have certain limitations. The median price was collected from 25 dental clinics in Kuala Lumpur, Malaysia, and the study was conducted in a single dental center, which might limit the generalizability of the results. The sample size was calculated with the assumption that a proportion of 60% of the subjects would be willing to pay the median price for the tooth replacement with implants. However, our results showed that only 30% were willing to pay the median price. Hence, a larger sample size consisting of different patient groups could provide us with greater insight into different factors that could affect the willingness to pay and serve as a useful guideline to establish an appropriate pricing guideline for dental implants.

## 5. Conclusions

The median price for dental implant treatment in Kuala Lumpur is RM 6000 (USD 1400). Annual income and personality traits are significant factors that may influence the willingness to pay for a single tooth implant. Patient education strategies for single tooth replacements with dental implants should be customized based on a patient’s personality and income to maximize effectiveness.

## Figures and Tables

**Table 1:** Patient demographics.

Patient Demographics		Group EV * (n = 50)	Group C * (n = 50)	*p* Value
Age (Mean (SD))		53.12 (12.51)	46.88 (10.82)	0.009
Gender	Male	23 (46%)	22 (44%)	0.841 (χ^2^ test)
	Female	27 (54%)	28 (56%)	
Basic educations	Comprehensive School	36 (72%)	36 (72%)	0.595 (χ^2^ test)
	Matriculation and University	14 (28%)	14 (28%)	
Professional status	Entrepreneur	13 (26%)	9 (18%)	0.257 (χ^2^ test)
	Upper CE	8 (16%)	10 (20%)	
	Lower CE	4 (8%)	5 (10%)	
	Worker	5 (10%)	12 (24%)	
	Student and Others	20 (40%)	14 (28%)	
Annual income	<RM 50,000 (12,000 USD)	17 (34%)	17 (34%)	0.758 (χ^2^ test)
	RM 50,000 (12,000 USD)–RM100,000 (24,000 USD)	20 (40%)	17 (34%)	
	>RM 1,000,000 (24,000 USD)	13 (26%)	16 (32%)	
Personality traits	Openness	26.22 (4.50)	26.92 (4.98)	0.843 (−2.587, 1.187) (*t*-test)
	Conscientiousness	21.34 (4.49)	20.58 (4.32)	0.577 (−0.991, 2.551) (*t*-test)
	Extraversion	19.94 (5.32)	19.24 (5.01)	0.629 (−1.352, 2.752) (*t*-test)
	Agreeableness	21.36 (6.98)	20.28 (4.71)	0.605 (−1.286, 3.446) (*t*-test)
	Neuroticism	27.08 (5.07)	26.86 (3.82)	0.042 (−1.563, 2.003) (*t*-test)

* C = Conventional, EV = Additional Evidence-based Video.

**Table 2:** Association of variables to willingness to pay the median price.

Patient Demographics		WTP the Median Price		*p*-Value (χ^2^ Test)
		Yes	No	
Gender	Male	17 (54.8%)	28 (40.6%)	0.19
	Female	14 (45.2%)	41 (59.4%)	
Age group	24–40	6 (19.4%)	17 (24.6%)	0.37
	41–50	6 (19.4%)	17 (24.6%)	
	51–56	7 (22.6%)	20 (29%)	
	57–83	12 (38.7%)	15 (21.7%)	
Basic Education	Comprehensive School	20 (64.5%)	52 (75.4%)	0.26
	Matriculation and University	11 (35.5%)	17 (24.6%)	
Professional Status	Entrepreneur	7 (22.6%)	15 (21.7%)	0.36
	Upper CE	8 (25.8%)	10 (14.5%)	
	Lower CE	2 (6.5%)	7 (10.1%)	
	Worker	3 (9.7%)	14 (20.3%)	
	Student and Others	11 (35.5%)	23 (33.3%)	
Annual income	<50,000 (12,000 USD)	6 (19.4%)	28 (40.6%)	0.03
	50,000 (12,000 USD–100,000 (24,000 USD)	11(35.5%)	26 (37.7%)	
	>100,000 (24,000 USD)	14(45.2%)	15 (21.7%)	
Intervention groups	Group C *	16 (32%)	34 (68%)	0.82
	Group EV *	15 (30%)	35 (70%)	

* C = Conventional, EV = Additional Evidence-based Video.

**Table 3:** Correlation of personality traits with the highest price subjects are willing to pay.

Variables	Mean (SD)	Pearson Correlations					
		WTP (Highest Price)	Openness	Conscientiousness	Extraversion	Agreeableness	Neuroticism
WTP (Highest price)	3737.5 (3296.4)	1.00	−0.25 *	−0.30 **	−0.12	−0.20 *	0.08
Openness	26.57 (4.74)	−0.25 *	1	0.34 **	0.45 **	0.22 *	−0.12
Conscientiousness	20.96 (4.41)	−0.30 **	0.34 **	1.00	0.48 **	0.40 **	−0.31 **
Extraversion	19.59 (5.16)	−0.12	0.45 **	0.48 **	1.00	0.18	−0.20 *
Agreeableness	20.82 (5.96)	−0.20 *	0.22 *	0.40 **	0.18	1.00	−0.48 **
Neuroticism	26.97 (4.47)	0.08	−0.12	−0.31 **	−0.20 *	−0.48 **	1.00
Age	50 (12.05)	0.13	0.01	−0.13	−0.14	−0.26 **	0.09

* Correlation is significant at the 0.05 level (2-tailed), ** Correlation is significant at the 0.01 level (2-tailed).

## Data Availability

The data presented in this study are available on reasonable request from the corresponding author.

## Supplementary Materials

The following are available online at https://www.mdpi.com/article/10.3390/healthcare9080952/s1, Table S1. Questionnaire on demographic factors, Table S2. Questionnaire to assess personality traits (OCEAN model), Table S3. Price charged for a dental implant from 25 dental clinics in Kuala Lumpur, Table S4. Association of Conscientiousness with willingness to pay the median price, Table S5. Maximum or minimum price that patients are willing to pay for a single tooth implant.
