# Supplementary material for: Personality Traits and Annual Income Determine the Willingness to Pay for a Single Tooth Implant
Source: Healthcare (Basel). 2021 Jul 29;9(8):952. doi: 10.3390/healthcare9080952 (PMC8391254; doi:10.3390/healthcare9080952)
Supplement: Supplementary file 1 [file healthcare-09-00952-s001.zip › healthcare-1294819-supplementary.pdf]

Supplementary Material

Personality traits and income determine the willingness to pay for a single tooth implant

Shirlene Foo Yih Ting[1#], Kimberley Chew Wen Chien[1#], Nurul Haniz Binti Ramzi[2], Allan Pau Kah Heng[1], Rohit Kunnath Menon[1*]

Affiliations:

[1]School of Dentistry, International Medical University, 57000 Kuala Lumpur, Malaysia

[2]Lecturer, Institute for Research, Development and Innovation, International Medical University, 57000 Kuala Lumpur, Malaysia

[#] Equal contribution

* Corresponding author

Table S1. Questionnaire on demographic factors

1. Gender
   Male
   Female

2. What is your basic education received?
   Comprehensive school
   Matriculation

3. Professional training
   Vocational qualification (school level)
   Vocational qualification (technical college)
   University or equivalent educational level

4. What is your current working situation?
   Working
   Not working

5. What is your professional status?
   Entrepreneur

Upper clerical employee
Lower clerical employee
Worker
Student
Other ___________

6. Annual income
< RM 50000
RM 50000 - 100000
> RM 100000

7. What is your year of birth?
____

8. Where was your last dental visit?
Private clinic
Public sector
Elsewhere

9. What would be the highest price you would be prepared to pay to replace a single tooth?
____

Table S2. Questionnaire to assess personality traits (OCEAN model)

Part A Openness to Experience

	Strongly Agree	Agree	Neutral	Disagree	Strongly Disagree
I am curious about many things					
I always come up with new ideas.					
I am creative and a deep thinker					
I have an active imagination					
I am inventive					
I value artistic experiences					
I prefer work that is routine					
I like to reflect and play with ideas					

I have few artistic interest					
I am advanced in art, music or literature					

Part B Conscientiousness

	Strongly Agree	Agree	Neutral	Disagree	Strongly Disagree
I can be somewhat careless					
I carry out my job thoroughly					
I am a reliable worker					
I tend to be disorganized					
I tend to be lazy					
I persevere until the task is finished					
I do things efficiently					
I make plans and follow through with them					
I am easily distracted					

Part C Extraversion

	Strongly Agree	Agree	Neutral	Disagree	Strongly Disagree
I see myself as someone who is reserved.					
I am talkative					
I am full of energy					
I tend to be quiet					
I have an assertive personality					
I am sometimes shy and inhibited					
I am outgoing and sociable					

Part D Agreeableness

	Strongly Agree	Agree	Neutral	Disagree	Strongly Disagree
I am helpful and unselfish with others					
I tend to find fault with others.					
I tend to start quarrel with others.					
I have a forgiving nature					
I am generally trusting					
I can be cold and isolated.					
I am considerate and kind to almost everyone.					
I am sometimes rude to others.					
I like to cooperate with others.					

Part E Neuroticism

	Strongly Agree	Agree	Neutral	Disagree	Strongly Disagree
I am relaxed and can handle stress well.					
I am depressed and blue					
I can be tense					
I worry a lot					
I am emotionally stable and not easily upset					
I can be moody					
I remain calm in tense situation					
I get nervous easily					

Table S3. Price charged for a dental implant from 25 dental clinics in Kuala Lumpur

1	RM 5500
2	RM 5500
3	RM 5900
4	RM 5999
5	RM 6000
6	RM 6000
7	RM 6000
8	RM 6000
9	RM 6000
10	RM 6000
11	RM 6000
12	RM 6000
13	**RM 6000 Median**
14	RM 6000
15	RM 6500
16	RM 7000
17	RM 7000
18	RM 7000
19	RM 7000
20	RM 7000
21	RM 7000
22	RM 7500
23	RM 7500
24	RM 8000
25	RM 9500

Table S4. Association of Conscientiousness with willingness to pay the median price

**Variable**	**Personality constructs**	**F**	**Sig**
**Willingness to pay the median price (Yes/No)**	Openness	1.731	0.192
	Conscientiousness	6.838	**0.01**
	Extraversion	0.737	0.393
	Agreeableness	1.153	0.286
	Neuroticism	0.797	0.374

Table S5. Maximum or minimum price that patients are willing to pay for a single tooth implant

Patient no:	Price (RM)
1	1500
2	8000
3	1000
4	7200
5	3000
6	3000
7	6000
8	2000
9	6400
10	2000
11	1000
12	1500
13	6000
14	10000
15	9900
16	1000
17	3000
18	1000
19	6000
20	10000
21	500
22	2000
23	2000
24	10000
25	1000
26	3000
27	7000
28	2000
29	8000
30	6000
31	6000
32	10000
33	6000
34	10000
35	15000
36	6000
37	4500
38	7000
39	10000
40	3000
41	1000
42	3000
43	5000
44	3000
45	1000
46	900
47	6000
48	1000
49	600

50	4000
51	1500
52	1000
53	4000
54	3000
55	10000
56	1500
57	1000
58	2000
59	2000
60	4000
61	1000
62	3000
63	10000
64	2000
65	2000
66	1500
67	1000
68	100
69	100
70	250
71	1000
72	2000
73	4000
74	500
75	6000
76	4000
77	5000
78	500
79	1000
80	1000
81	1000
82	3500
83	6000
84	500
85	6000
86	500
87	300
88	10000
89	2000
90	6000
91	2000
92	2000
93	500
94	1000
95	4000
96	2000
97	1000
98	12000
99	2500
100	1000
